# Linking the potato genome to the conserved ortholog set (COS) markers

**DOI:** 10.1186/1471-2156-14-51

**Published:** 2013-06-08

**Authors:** Hannele Lindqvist-Kreuze, Kwangsoo Cho, Leticia Portal, Flor Rodríguez, Reinhard Simon, Lukas A Mueller, David M Spooner, Merideth Bonierbale

**Affiliations:** 1 International Potato Center, Lima, Peru; 2 USDA-Agricultural Research Service, Vegetable Crops Research Unit, University of Wisconsin, Madison, WI, USA; 3 Boyce Thompson Institute for Plant Research, Ithaca, NY, USA; 4 Current address: Highland Agriculture Research Center, Rural Development Administration, Pyeongchang, South Korea

**Keywords:** Conserved ortholog set (COS), Genetic mapping, Potato genome, *Solanum*

## Abstract

**Background:**

Conserved ortholog set (COS) markers are an important functional genomics resource that has greatly improved orthology detection in Asterid species. A comprehensive list of these markers is available at Sol Genomics Network (http://solgenomics.net/) and many of these have been placed on the genetic maps of a number of solanaceous species.

**Results:**

We amplified over 300 COS markers from eight potato accessions involving two diploid landraces of *Solanum tuberosum* Andigenum group (formerly classified as *S. goniocalyx*, *S*. *phureja*), and a dihaploid clone derived from a modern tetraploid cultivar of *S. tuberosum* and the wild species *S. berthaultii*, *S. chomatophilum*, and *S. paucissectum*. By BLASTn (Basic Local Alignment Search Tool of the NCBI, National Center for Biotechnology Information) algorithm we mapped the DNA sequences of these markers into the potato genome sequence. Additionally, we mapped a subset of these markers genetically in potato and present a comparison between the physical and genetic locations of these markers in potato and in comparison with the genetic location in tomato. We found that most of the COS markers are single-copy in the reference genome of potato and that the genetic location in tomato and physical location in potato sequence are mostly in agreement. However, we did find some COS markers that are present in multiple copies and those that map in unexpected locations. Sequence comparisons between species show that some of these markers may be paralogs.

**Conclusions:**

The sequence-based physical map becomes helpful in identification of markers for traits of interest thereby reducing the number of markers to be tested for applications like marker assisted selection, diversity, and phylogenetic studies.

## Background

The use of genetic diversity in plant breeding is a sustainable method to conserve valuable genetic resources and to increase agricultural productivity and food security [1]. To facilitate the use of the wide genetic diversity existing in landraces and crop wild relatives more information is needed on the organization and structure of their genes and genomes. Molecular markers linked to loci with important effects hold a promise to facilitate the introgression of those traits into adapted germplasm. Agriculturally important traits captured during domestication are often coded by very limited number of loci with major phenotypic effects. Within the Solanaceae it is common to find that these loci have putative orthologous counterparts in other species [2] and therefore molecular markers, such as Conserved Orthologous Set (COS) markers, are powerful in comparing genomic information across species [3].

The development of markers for orthologous genes, many of which have been mapped in tomato, is documented in the Sol Genomics Network [4]. Comparative mapping studies with the help of COS markers have shown syntenic relationships within various species of the Solanaceae family [5-7] and between species within the Asterid and Rosid clades comparing coffee (Rubiaceae, Asterid) with tomato (Solanaceae, Asterid) [8] and coffee and grapevine (Vitaceae, Rosid) [9]. The combined power of comparative mapping and systematic analysis of germplasm with orthologous gene markers can efficiently leverage information generated by genomic research from one species to another. COS markers also have shown great power in resolving interrelationships of tomato and potato with great precision [10].

The recent accumulation of nucleotide sequences of model organisms and crop plants has provided fundamental information for the design of sequence-based research applications in functional genomics [11]. The draft genome sequence of potato has been publicly available since late 2010 and the finalized high-quality sequence has been released [12] as well as the genome sequence of closely related tomato [13]. The availability of these genomes and the genomic tool kits, such as genome browsers, are of great importance to the scientific community working with solanaceous crops. With the help of physical sequences, new molecular markers can be developed efficiently, utilizing genes in the regions of the genome that contain markers linked to traits of interest. The possibility of comparing physical and genetic maps also has implications for molecular breeding programs, facilitating the search of molecular markers flanking QTL [14]. Linking COS markers to the potato genome sequence allows for powerful comparative genomics between the potato genome and other species with COS-based maps that do not yet have genome sequence available.

Here we present a case study where COS are amplified from diverse set of *Solanum* germplasm and aligned to the whole genome sequence of potato, allowing for comparison of physical and genetic maps of related species. We aligned the sequences of COS, generated from a panel of ten genotypes of potato and tomato, to the recently published potato genome sequence and compared the physical location with the genetic location in tomato and potato. We show that the COS markers analyzed are single- or low-copy in the DM potato genome (see Methods) and that there are several breaks in co-linearity between the species analyzed.

## Results

### *In silico* mapping of COS sequences into the potato genome

In total, 322 COS were mapped *in silico* in the potato genome, from here on referred to as DM, utilizing the DM superscaffold sequences (Additional file 1: Table S1). To verify that the hits located inside predicted genes, we ran BLASTn against the DM gene sequences and found that ten COS had no matching DM gene although they had high confidence hits in the superscaffold sequence; we did not pursue these markers further. The COS markers are distributed throughout the genome (Additional file 1: Table S1) and the majority exist as single copy markers. However, 17 markers are present in multiple copies (Table 1) with either existing in tandem repeats in the same genome region or having copies in different genomic regions. After single copy, the most frequent copy number is two and the highest copy number is three.

**Table 1:** COS markers with multiple hits in DM superscaffolds and their corresponding DM gene hits

**COS**	**Genetic map chromosome**	**DM gene**	**Best evalue**	**DM chromosome**	**DM gene annotation**
T0408	11	PGSC0003DMG400029022	0E	11	Aminotransferase
		PGSC0003DMG400046906	0E	1	Gene of unknown function
At5g27620	1, 4	PGSC0003DMG400024698	9E-39	1	CAK associated cycling H homolog
		PGSC0003DMG400009473	1E-155	4	
At3g63200	9	PGSC0003DMG400012878	0E	9	Patatin T5
		PGSC0003DMG400020128	0E	6	
At2g41680	9	PGSC0003DMG402027256	0E	4	Thioredoxin reductase
		PGSC0003DMG400023691	0E	10	
At1g29260	na	PGSC0003DMG400008719	0E	3	Peroxisomal targeting signal type 2 receptor
		PGSC0003DMG400009000	0E	1	
At1g10580	2	PGSC0003DMG402030529	0E	5	Conserved gene of unknown function
		PGSC0003DMG400022364	0E	2	
At1g08550	na	PGSC0003DMG400020993	9E-59	4	Violaxanthin epoxidase
		PGSC0003DMG400010690	5E-51	4	
		PGSC0003DMG400010688	3E-71	4	
At1g14980	5	PGSC0003DMG402023448	1E-99	5	small molecular heat shock protein
		PGSC0003DMG400028744	1E-110	7	CPM10 protein
At2g42620	12	PGSC0003DMG400007856	0E	12	F-box family protein
		PGSC0003DMG400035320	1E-112	7	F-box/leucine rich repeat protein
At2g46370	10	PGSC0003DMG401000095	0E	1	Jasmonic acid amino acid conjugating enzyme
		PGSC0003DMG400033879	3E-92	5	
At3g19895	4	PGSC0003DMG400032542	0E	4	Conserved gene of unknown function
		PGSC0003DMG400019001	1E-105	11	
At4g11820	8	PGSC0003DMG400004253	1E-124	12	HMG CoA synthase
		PGSC0003DMG400022749	1E-74	8	
		PGSC0003DMG400022750	1E-110	8	
At4g24620	4	PGSC0003DMG400030128	9E-72	1	Glucose 6 phosphate isomerase
		PGSC0003DMG400012910	1E-107	4	
		PGSC0003DMG400009848	1E-77	na	
At5g20890	11	PGSC0003DMG400019637	0E	11	chaperonin containing T-complex protein 1, beta subunit
		PGSC0003DMG400007161	9E-54	6	T-complex protein 1 subunit beta
		PGSC0003DMG400012727	0E	4	
T0805	6	PGSC0003DMG400020484	1E-158	6	ATP synthase subunit b’ chloroplastic
		PGSC0003DMG400020466	1E-158	6	
T0989	7, 12	PGSC0003DMG402013561	0E	7	Pyruvate dehydrogenase E1 alpha subunit
		PGSC0003DMG400002921	1E-167	12	
T1511	3	PGSC0003DMG400018190	1E-160	3	Elongation factor TuA
		PGSC0003DMG400041767	1E-63	6	Elongation factor TuB

### Genetic linkage maps

For genetic mapping in potato we utilized mostly the back cross progeny BCT [15]. 186 COS markers were placed on the BCT consensus linkage map, which contains in total 321 markers assembled into 12 linkage groups. The total length of the consensus BCT map was 1042 cM, the average marker interval was 3.4 cM and the maximum interval was 34.7 cM on chromosome 12. In addition three COS markers were integrated on the BCT paternal map because they would not integrate on the consensus map.19 markers that were not polymorphic in the BCT parents, were placed on the previously published frame work genetic maps of PCC1 [16] and PD [17]. The genetic maps are shown in Additional file 2: Table S2.

### Comparison between *in silico* and genetic maps

A total of 208 COS were placed on the potato genetic maps (Additional file 1: Table S1). Of these, 173 were also mapped *in silico*, but there are 35 markers that were only mapped genetically because their DNA sequences were not available. The Tomato EXPEN2000 genetic map, from here on referred to as TomEXPEN, was used as a reference and the map locations of the COS markers *in silico* mapped in potato in this project were downloaded from the SGN web site [4]. Of the 322 COS mapped *in silico* 254 were found in the TomEXPEN map.

**Figure 1:**
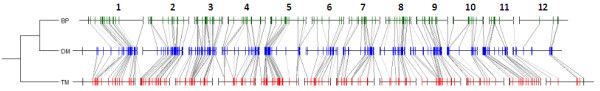
**Comparative map of the potato genetic map (BP: integrated map of BCT, PD and PCC1), the potato genome (DM) and the tomato genome (TM). **The potato genetic map was scaled to the size of the corresponding DM pseudomolecule setting the last COS marker of each linkage group equal to the size of the pseudomolecule. Likewise, the tomato genetic map was scaled using the pseudomolecule size of the corresponding tomato physical map. Lines are drawn between corresponding COSII markers. A generic tree is drawn to the left hand grouping visually the two potato maps versus the tomato map. Linkage groups and pseudomolecules are drawn sequentially from left to right as indicated by the numbers.

Based on the previous information on their location in the TomEXPEN map, most of the COS markers mapped into the expected potato chromosomes either in the reference potato genome, (DM) or in the potato genetic maps BCT, PCC1 or PD (BP) (see Methods; Figure 1). Of the 173 shared markers between DM and the potato genetic maps, eight map in different chromosomes and 12 have one copy mapping on the same chromosomes and a second copy in another one (Additional file 1: Table S1). Of the 254 markers shared by DM and TomEXPEN, ten are in different chromosomes and nine have one copy mapping in the same chromosomes and a second copy in another chromosome. Of the 305 markers that had a single location in DM, in total 15 mapped in unexpected chromosomes when compared to tomato or potato genetic maps (Table 2). These markers had a single matching DM gene hit except for two markers which had no gene hit. The difference may be a real one suggesting major differences in genome organization but it may also reflect errors in sequence assembly or genetic mapping.

**Table 2:** DM genes corresponding to the single copy COS markers that map in unexpected chromosomes

**COS marker**	**DM gene**	**e-value**	**DM chromosome**	**Genetic map**	
At1g28380	PGSC0003DMG400031748	1.00E-169	2	8, 2	b
At1g65720	PGSC0003DMG400024083	0	3	2	a
At1g67740	PGSC0003DMG400007201	0	10	4	b
At1g77290	PGSC0003DMG400025321	0	4	6, 4	b
At3g44890	-No-Hit-		12	11	a
At4g26180	PGSC0003DMG400000342	1.00E-150	12	10	b
At5g04270	PGSC0003DMG400023477	1.00E-117	5	11	b
At5g06760	PGSC0003DMG400011439	5.00E-99	10	1	a
At5g08580	PGSC0003DMG400003301	1.00E-87	10	2	a, b
At5g60940	-No-Hit-		12	8	b
At5g64730	PGSC0003DMG400016731	1.00E-102	11	12	b
T0393	PGSC0003DMG400022210	0	7	9	a
T0966	PGSC0003DMG400014388	0	10	7, 10	a
T0974	PGSC0003DMG400004283	0	12	4	a
T1391	PGSC0003DMG400031021	4.00E-60	2	1, 10	a, b

a TomEXPEN2000 map.

b BCT, PCC1 or PCC1 map.

Markers having unexpected locations were found in all chromosomes, but the highest number of these was in chromosome 10. Pairwise comparisons between the three maps show that eight markers that locate in chromosome 10 in at least one of the maps have an alternative locus in another chromosome (Additional file 1: Table S1). These markers are: C2_At2g46370, (*in silico* 1 and 5, tomato 10); C2_At3g60080 (*in silico* 2, tomato 10); T1391 (*in silico* 2, potato 1 and 10, tomato 10); T0966 (*in silico* 10, potato 10, tomato 7); C2_At5g08580 (*in silico* 10,potato 2, tomato 2); C2_At5g06760 (*in silico* 10, tomato 1); C2_At4g26180 (*in silico* 12, potato 10, tomato 12); C2_At2g41680 (*in silico* 4 and 10, in tomato 9). Differences are mostly specific to the genetic maps, meaning that the marker position is usually conserved in two of the maps. Also, multi-copy markers mapping to different chromosomes *in silico* in DM are mostly found in one of the same chromosomes in the genetic maps. For example, marker C2_At2g42620 in DM maps in chromosomes 12 and 7, whereas in tomato it only maps in chromosome 12. This could be simply because the alternative marker was not detected due to lack of polymorphism or because the other sequence detected by BLASTn search is a paralog.

The COS that mapped in the same chromosomes by both methods (*in silico* in potato and genetically in potato or in tomato) as found at SGN were not always in agreement in their exact order, reflecting errors either in statistical testing or differences between the solanaceous species at the microsynteny level. In addition to the nine large inversions between tomato and potato several small inversions have been demonstrated [13]. In total, 77 COS that were mapped in potato (either *in silico* or genetically) were not found on the TomEXPEN map and thus we were not able to compare their locations.

### Multiple copy markers

**Figure 2:**
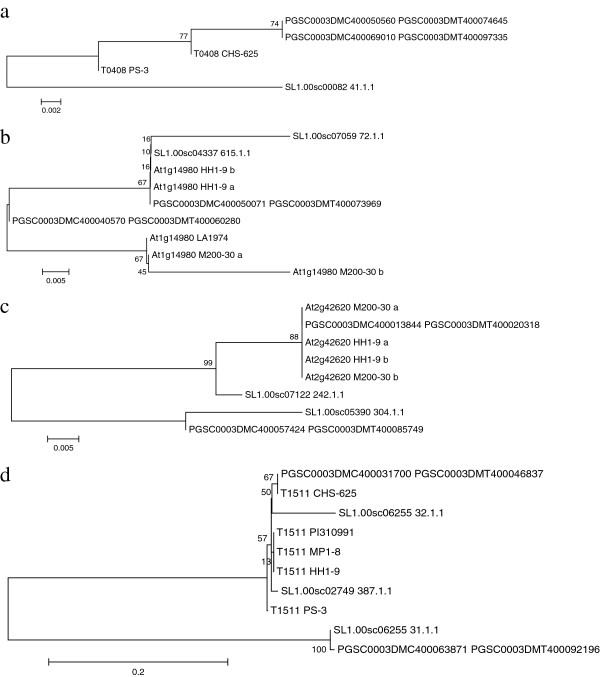
**Evolutionary relationships of the COS marker sequences and the corresponding DM gene sequences inferred by Neighbor Joining (NJ) analysis. **NJ trees for markers T0408 (222 sites) (**a**), At1g14980 (39 sites) (**b**) and At2g29260 (112 sites) (**c**) were constructed from translated amino acid sequences. The evolutionary distances were computed using the Poisson correction method and are in the units of the number of amino acid substitutions per site. Tree for marker T1511 (256 sites) (**d**) is from nucleotide sequence. The evolutionary distances were computed using the Maximum Composite Likelihood method and are in the units of the number of base substitutions per site. The percentage of replicate trees in which the associated taxa clustered together in the bootstrap test (1000 replicates) are shown next to the branches.

We observed 17 markers that were duplicated in the potato genome. To find the DM genes that correspond to these markers we ran a BLASTn search against the Potato Genome Sequencing Consortium (PGSC) databases containing the genes and the coding sequences. For most of the markers (in total 13) all copies had the same annotation suggesting that they could be orthologs (Table 1). The four markers that have different annotations for the copies are T0408, At1g14980, At2g42620 and T1511. To further test the ortholog/paralog relationship of these markers we aligned the potato and tomato reference genome gene sequences, coding sequences and query sequences for these markers and constructed Neighbor Joining trees (Figure 2).

T0408 marker was sequenced from two genotypes, the parents of the PD population (CHS_625 and PS-3). This marker is entirely in the exon region and is similar to the genes PGSC0003DMG400046906 (gene of unknown function) on chromosome 1 and PGSC0003DMG400029022 (aminotransferase) in chromosome 11 (Table 1). In the TomEXPEN map this marker is found in chromosome 1. The coding sequences PGSC0003DMC400069010 and PGSC0003DMC400050560 are identical in the query sequence region consisting of 119 amino acids. However, outside this area the two DM CDS are not identical. Genotype CHS-625 differs from the DM sequences in only one amino acid. Genotype PS-3 is highly heterozygous and because only one sample was sequenced and we cannot resolve the two possible haplotypes of this genotype and therefore it appears different from the rest of the sequences (Figure 2a). The corresponding tomato reference genome coding sequence is quite different from the potato sequences. In this case the gene may be single copy but the marker may be unspecific, resulting in alternative hits.

Marker At1g14980 was amplified from genotypes LA1974, HH1-9 and M200-30 and the sequences are similar to PGSC0003DMG400028744 (PGS0003DMC400050071) in chromosome 7 and PGSC0003DMG402023448 (PGSC0003DMC400040570) in chromosome 5 with the e values of 1.00E-110 and 1.00E-99, respectively. The marker spans both exonic and intronic regions. Translated amino acid sequences of the exonic regions show two well resolved groups where two sequences from M200-30 group together with one of the tomato genomic sequences and two sequences from HH1-9 group with the CDS of the gene that maps in chromosome 5. Relationships with the other DM coding sequence are not well resolved (Figure 2b). Genetic mapping in potato suggests that the marker resides in chromosome 5. However, based on the sequence data we cannot determine the correct location for this marker.

Marker At2g42620 sequences from the BCT population parents (HH1-9 and M200-30) have hits in genes PGSC0003DMG400007856 (F-box family protein) and PGSC0003DMG400035320 (F-box/leucine rich repeat protein) with the e values of 0 and 1.00E-112, respectively. The first gene is found in chromosome 12 and the latter in chromosome 7. According to the NJ tree, all our sequences from the genotypes HH1-9 and M200-30 are more similar to the first mentioned gene represented by the coding sequence PGSC0003DMC400013844 (Figure 2c). The latter DM gene has some amino acid changes comparing with the others and thus may code for a different gene as already shown by the different annotations (Table 1). Genetically this marker is found in chromosome 12 in tomato which most likely is its correct location.

Marker T1511 was amplified from five genotypes (CHS-625, PS-3, PI310991, MP1-8, and HH1-9). According to the BLASTn analysis it is similar to the DM genes PGSC0003DMG400018190 (Elongation factor TuA) in chromosome 3 (1E-160) and PGSC0003DMG400041767 Elongation factor TuB, 6E-63) in chromosome 6. In NJ tree all genotypes are more closely related to the first gene represented by the CDS PGSC0003DMC400031700 (Figure 2d). The marker resides in the exon and has quite variable sequence even at the amino acid level. Because this marker has been genetically mapped in chromosome 3 in tomato and the evalue for the hit in chromosome 3 is higher (Table 1), this is most likely its correct location. Of the three corresponding tomato coding sequences, two group with the chromosome 3 gene.

A comparative summary of the maps is shown in Figure 1. Overall the alignment of COSII markers follows a sequential order. However, as described above several COSII markers show differences as indicated by crossing lines or lines indicating locations on different linkage groups or pseudomolecules.

### COS markers with a putative function and QTL for late blight resistance and vitamin synthesis

There is a large overlap of QTL regions between the traits included and based on this information alone the same markers may be considered candidates for disease resistance and Carotenoid or vitamin C biosynthesis (Additional file 1: Table S1 and Additional file 3: Table S3). Therefore, functional annotations of the matching DM genes (Additional file 3: Table S3) may help suggesting markers in candidate genes for the QTL traits and for further studies.

### Ontology term annotation analysis

The SEA analysis showed that the COSII-DM list contained no associated ontology terms that were significantly different in the biological process gene ontology category as compared to the list of terms associated with the original COSII list. However, both the COSII-DM and the original COSII term list have associated term lists that are enriched for 33 GO terms in the biological process category that is different (see Table 3 and Additional file 4: Figure S1). The terms form two major groups: a) cellular metabolic process and b) response to stimulus.

**Table 3:** Significantly enriched terms in the biological process category of the gene ontology associated with COSII markers mapped onto the DM genome

**GO term**	**Description**	**Number in COSII-DM list**	**Number in TAIR9 list**	**p-value**	**FDR**
GO:0009108	coenzyme biosynthetic process	8	98	6.6E-07	0.00061
GO:0051188	cofactor biosynthetic process	10	191	1.7E-06	0.00079
GO:0043436	oxoacid metabolic process	20	859	0.000009	0.0011
GO:0034641	cellular nitrogen compound metabolic process	15	506	6.9E-06	0.0011
GO:0009628	response to abiotic stimulus	28	1471	8.6E-06	0.0011
GO:0006082	organic acid metabolic process	20	860	9.2E-06	0.0011
GO:0019752	carboxylic acid metabolic process	20	859	0.000009	0.0011
GO:0042180	cellular ketone metabolic process	21	882	0.000004	0.0011
GO:0006519	cellular amino acid and derivative metabolic process	17	682	0.000017	0.0017
GO:0051186	cofactor metabolic process	11	308	0.00002	0.0018
GO:0006520	cellular amino acid metabolic process	13	430	0.000022	0.0018
GO:0044106	cellular amine metabolic process	13	438	0.000027	0.002
GO:0009308	amine metabolic process	14	521	0.000039	0.0028
GO:0006732	coenzyme metabolic process	8	188	0.000078	0.0052
GO:0009651	response to salt stress	11	366	0.000094	0.0058
GO:0050896	response to stimulus	52	4057	0.00017	0.0098
GO:0008152	metabolic process	114	10614	0.0002	0.011
GO:0010035	response to inorganic substance	9	279	0.00023	0.011
GO:0009657	plastid organization	6	119	0.00024	0.011
GO:0006970	response to osmotic stress	11	408	0.00024	0.011
GO:0046686	response to cadmium ion	7	178	0.00035	0.015
GO:0044237	cellular metabolic process	95	8722	0.00034	0.015
GO:0010038	response to metal ion	8	238	0.00039	0.016
GO:0006461	protein complex assembly	6	134	0.00046	0.017
GO:0070271	protein complex biogenesis	6	134	0.00046	0.017
GO:0006629	lipid metabolic process	16	841	0.00058	0.021
GO:0016054	organic acid catabolic process	5	98	0.00075	0.025
GO:0046395	carboxylic acid catabolic process	5	98	0.00075	0.025
GO:0009791	post-embryonic development	14	705	0.00082	0.026
GO:0051641	cellular localization	12	569	0.0011	0.034
GO:0015979	photosynthesis	6	162	0.0012	0.037
GO:0006950	response to stress	31	2320	0.0014	0.039
GO:0044272	sulfur compound biosynthetic process	5	115	0.0015	0.043

## Discussion

COSII markers represent an important functional genomics resource that has greatly improved comparative mapping in Asterid species. They can be used to design primer sequences for cleaved amplified polymorphic sequence (CAPS) useful for genetic mapping across diverse taxa, including the Solanaceae. In genetic mapping, the number of markers placed on the map is dependent on the number of polymorphisms between the parents of the cross. Our initial goal, before the availability of the genome sequence, was to facilitate comparative mapping in the Solanaceae by mapping 300 single-copy COSII in potato, *Solanum tuberosum*, to a diploid mapping population. However, limitations mostly in the level of polymorphism resulted in the successful genetic mapping of only 208 markers using three different segregating populations. The availability of the potato genome sequence enabled another approach to be taken to investigate the genomic locations of these markers in potato. With the help of BLAST analysis we successfully mapped over 300 orthologous markers *in silico* and compared their physical location in the reference potato genome to that of the genetic location in a potato cross and in previously published map of tomato. Because we utilized DNA sequences obtained from various *Solanum* species we were able to sample some of the polymorphism present in these taxa and thereby detect markers that are potentially present in multiple copies. We found that most of the markers are present as single-copy in the reference genome. Low copy number is a required character for markers intended for comparative genetic mapping and phylogenetic analysis. Low-copy sequences generally evolve independently of paralogous sequences and tend to be stable in position and copy number. However, a potential problem is the existence of gene families producing paralogs that can evolve independently [18] and the fact that some genes characterized as low-copy in some groups can be multiple copy in others. We discovered that very low number of the COS markers tested here (17 out of 354, 4.7%) were designed on genes that were present in multiple copies in potato, thus validating the low-copy number definition of these markers.

*In silico* mapping using the BLASTn algorithm seems to work well in mapping COS marker sequences into the reference genome. This is because the COS primers have been designed to amplify a PCR fragment in the size range that is suitable for BLAST and they have been tested through rigorous algorithms to target genes that are present in single or low-copy numbers [19]. The BLAST algorithm may result in the identification of paralogous sequences. This is a problem only in the case of incomplete reference sequence dataset or when the target genes belong to gene families. Since our input database is the complete genome sequence of potato and most of the markers resulted in a single hit in the genome it is likely that the genes identified are true orthologs. However, for the sequences resulting in multiple hits, it is necessary to make gene-level comparisons when attempting to distinguish paralogues from orthologs. For the markers that target intronic regions, this may be difficult.

The ontology enrichment analysis showed that no bias was introduced in the COSII-DM list as compared to the original COSII list. In general, both gene lists may have a slight overrepresentation of genes in cellular metabolic process and response to environmental stress, and be related to QTLs and agronomic traits of interest like yield, quality and resistance. Considering COS markers that locate in previously published QTL as candidate genes for a given trait may be difficult because the QTL regions span large parts of the chromosome. However, functional annotations are helpful in narrowing down to some specific candidate genes. Some obviously interesting candidate markers for late blight resistance are C2_At5g51840 (*Rar1*) and C2_At4g36530 (Cinnamoyl-CoA reductase) in chromosome 11 as well as C2_At4g02600 (MLO1) in chromosome 9. RAR1 is required for the functionality of several R genes [20], while Cinnamoyl-CoA reductase is the first enzyme on the pathway leading to production of Lignin, which is an important factor in plant defense responses and MLO1 confers broad spectrum mildew resistance in barley [21]. Obvious candidate markers for carotenoid and vitamin C biosynthesis are not that easy to identify from this study. However, the QTL regions for these traits contain a couple of photosynthesis and chloroplast related genes, which is to be expected since carotenoids function in photosynthesis acting as pigments in the light harvesting complexes and vitamin C is just a few biochemical steps away from ‘sugar’ produced by photosynthesis. Carotenoids have two key functions in plants: broaden the light spectrum for light harvesting and protecting the chlorophyll against oxidative damage or excess energy [22]. Overlapping regions for QTL for vitamin C biosynthesis and disease resistance are not surprising since many biological processes are altered in the plant during defense response. For example ascorbic acid content in leaves has been shown to modulate plant defense transcripts [23] and has been suggested to protect the cells against oxidative stress arising from wounding [24].

We found only a few COS markers that mapped in unexpected chromosomes. In cases where one copy was detected in the same chromosome as in the genetic map and an additional copy in an alternative locus, it is possible that one of the markers detected originates from a paralog. Often these can be readily detected by choosing the gene hit with the best e-value. The single copy markers that have unexpected locations between physical and genetic maps may be true differences as we are comparing different species (DM = phureja, BCT = berthaultii × tuberosum, PCC1 = paucissectum × chomatophilum, PD = phureja × tuberosum, and finally tomato). Tomato and potato are generally considered to be highly colinear in their gene order [13,25,26], and this is true for the majority of the RFLP markers shared by the tomato and potato maps at the SGN website [4]. According to Tanksley et al., [26] tomato and potato genomes differ by only five paracentric inversions while these two species differ from pepper and eggplant by many more complex rearrangements, mainly paracentric inversions and translocations [27,28]. According to the most recent tomato/potato comparison there are nine major inversions and several small ones [13]. Significant conservation is found between distantly related species from the Asterid (*Coffea canephora* and *Solanum* sp.) and Rosid (*Vitis vinifera*) clades, at the genome macrostructure and microstructure levels [9]. A minimum of three (and up to ten) inversions and 11 reciprocal translocations differentiate the tomato genome from that of the last common ancestor of *Nicotiana tomentosiformis* and *N*. *acuminata*[6].

It is possible that the potato reference sequence may contain small numbers of incorrectly oriented or misplaced scaffolds as well as genes that were not discovered by the gene prediction algorithm used. As seen in this work we found a number of markers that had a high confidence hit in the whole genome sequence, but no gene hit. We ran those genome regions through Softberry gene prediction and were able to identify genes matching the COS marker hit region (results not shown). Further work focusing on the genome regions that from this work show contradictory results may facilitate the refinement of the genome assembly and annotation.

The high degree of conservation of gene order (synteny) in the Solanaceae revealed by cross mapping of homologous gene sequences has provided insights into genome evolution and has enabled the cloning of genes for agronomically important traits [29,30]. However, when comparing two genetic maps it is necessary to take into account that the number of markers shared by any two maps is rather small, and therefore allows only a limited resolution for comparison. Recent comparisons of physical maps between solanaceous species have allowed for more detailed level of comparison of gene order and orientation [31,32]. Comparison of orthologous regions shows general colinearity between solanaceous species, but also local breaks due to inversions and/or indels. Also, some of the inconsistencies in sequential ordering may well be artifacts since both the potato and the tomato genome still contain scaffolds that could not be oriented. Our results may help to refine the assembly and annotation of the potato and tomato genome.

The distances between markers on a genetic linkage map are based on the proportion of recombination events occurring within a given chromosome segment and thus indicative of gene order at a much lower resolution than physical map distances, which are the actual nucleotide sequence based distances. The sequence-based physical map becomes helpful in identification of markers near traits of interest and thereby reducing the number of markers to be tested in developing applications such as marker assisted selection, diversity assessment, and phylogeny.

## Conclusions

The COS markers studied are mostly present as single copies in the reference potato genome sequence, making them ideal for applications such as diversity and phylogenetic studies. *In silico* mapping is complementary to genetic mapping and facilitates detailed marker identification for traits of interest.

## Methods

### Plant material

Parents of the BCT [15], PCC1 [16], PD [17], the DM/DI//DI (developed at CIP and contributed to the Potato Genome Sequencing Consortium for anchoring of the DM potato genome [33], and tomato mapping populations [34] were subjected to COS marker amplification intended for DNA sequencing. The progeny from BCT backcross population (M200-30 (USW2230 × PI473331) × HH1-9) involving *Solanum berthaultii* and S*. tuberosum*[15], PCC1 [16] and PD [17] were used for genetic mapping. In addition, COS were amplified from other asterid species *Ipomoea trifida* genotypes M9 (CIP107665.9) and M19 (CIP 107665.19), and *Daucus carota* genotypes QAL and 0493B [35] for cross species comparisons. Leaf tissue was ground in liquid nitrogen and genomic DNA was extracted using standard protocol [36].

### Marker detection

COS markers were selected comparing the published genetic maps with the tomato COS map [4] and selecting markers that located in the QTL intervals for late blight resistance and/or maturity [16,17,37-44], ascorbic acid biosynthesis [45] and carotenoid biosynthesis [46] (Additional file 1: Table S1). In addition markers with annotations to genes known to have function in abiotic and biotic stress were selected.

COS markers were amplified from genomic DNA and the optimal annealing temperature for each primer pair was determined using temperature gradient. PCR reactions were conducted with 25 ng of DNA in a 1× PCR buffer (10 mM tris HCl, pH 8.3, 50 mM KCl, 1.5 mM MgCl_2_, 0.1% Triton-X), 0.2 mM of each dNTP, 0.2 mM of each primer forward and reverse and 0.5 U of Taq polymerase. Reactions were set up in microplates and processed in an MJ Research model PTC-200 PCR thermocycler with the following cycles: 1 cycle at 94°C for 4 min, 35 cycles at 94°C for 1 min plus 55 or 60°C for 1 min plus 72°C for 1 min, and 1 cycle at 72°C for 5 min. The bands were separated by SSCP (single-stranded conformation polymorphism) electrophoresis using 6% denatured (7M urea) polyacrylamide (19:1) and visualized by silver staining. All well-separated bands were cut from the gels with a razor blade. The excised gel slices were placed on 96-well PCR plates, and the DNA was eluted in 40 uL of sterile nuclease free water. This was used as a template in a new PCR reaction with the same primers in a 10 uL reaction.

One μL of this product was sequenced with the same primers in a 5 μL reaction using the ABI Big Dye dideoxynucelotide termination kit (Applied Biosystems, Foster City, California). Amplifications were carried out in an MJ Research DNA Engine Dyad^®^ Peltier Thermal Cycler (Watertown, Massachusetts) using an initial denaturation at 95°C for 3 min, followed by 30 cycles of 96°C for 25 s, 50°C for 20 s, 60°C for 5 min and with a final elongation at 72°C for 7 min. Excess of dye terminators were removed using CleanSeq magnetic bead sequencing reaction clean up kit from Agencourt Biosciences (Beverly, MA). Sequences were resolved on an ABI 3730xl capillary-based automated DNA sequencer (Applied Biosystems) with 50 cm POP-7 polymer capillaries at the Biotechnology Center of the University of Wisconsin-Madison. Alternatively, for some of the markers the PCR products were isolated and purified with Qiaquick Gel Extraction kit and sequenced without the previous re-amplification step.

### Sequence data

Publicly available sequence files and other data of potato *S. tuberosum* Group Phureja DM1-3 516R44 (CIP801092) generated by the Potato Genome Sequencing Consortium were obtained from [47]. We used the v3 superscaffold sequences, v2.1.10 AGP Pseudomolecule Sequences, 3 DM Pseudomolecule AGP data (v2.1.10), v3.4 gene sequences, and v3.4 cds. Tomato genome sequences were obtained from [48]. We used the ITAG1 release cds and genomic sequences.

### *In silico* mapping

We used VectorNTI to assemble the COS marker DNA sequences and queried the consensus sequences of contigs formed by at least two sequences against the DM superscaffolds using BLASTn. The DNA sequences of the COS markers were deposited to the NCBI GenBank GSS database and SGN database (Table 4). The exact location of each COS in the DM genome was obtained by selecting the best matching hit location based on e-value. The positions of the COS in the DM physical map were determined with the help of the superscaffold location information in pseudomolecules according to the pseudomolecule report v.2.1.9 provided by PGSC.

**Table 4:** The names and accession codes of the COS marker DNA sequence libraries deposited in the NCBI GenBank GSS database

**Library accn#**	**Library name**
LIBGSS_038998	*Daucus carota* (0493B) genomic DNA extraction from leaf tissue
LIBGSS_038999	*Daucus carota* (QAL) genomic DNA extraction from leaf tissue
LIBGSS_039000	*Ipomoea trifida* (M19) genomic DNA extraction from leaf tissue
LIBGSS_039001	*Ipomoea trifida* (M9) genomic DNA extraction from leaf tissue
LIBGSS_039002	*Solanum chomatophilum* (PI310991-1) genomic DNA extraction from leaf tissue
LIBGSS_039003	*Solanum* hybrid (MP1-8) genomic DNA extraction from leaf tissue
LIBGSS_039004	*Solanum* hybrid (M200-30) genomic DNA extraction from leaf tissue
LIBGSS_039005	*Solanum lycopersicoides* (LA2951) genomic DNA extraction from leaf tissue
LIBGSS_039006	*Solanum sitiens* (LA1974) genomic DNA extraction from leaf tissue
LIBGSS_039007	*Solanum tuberosum* (PS-3) genomic DNA extraction from leaf tissue
LIBGSS_039008	*Solanum tuberosum* (HH1-9) genomic DNA extraction from leaf tissue
LIBGSS_039009	*Solanum tuberosum* (CHS-625) genomic DNA extraction from leaf tissue
LIBGSS_039010	*Solanum tubersoum* (DI) genomic DNA extraction from leaf tissue
LIBGSS_039011	*Solanum tuberosum* (DMDI) genomic DNA extraction from leaf tissue

### Genetic mapping

Three diploid mapping populations BCT [15], PCC1 [16] and PD [17] were used for segregation analysis to locate COS in potato linkage groups. Polymorphisms were detected by high resolution melting (HRM), [49], SSCP followed by silver staining or by agarose gel electrophoresis. For HRM the PCR amplification was performed with the fluorescent DNA-binding dye (LCGreen) and the DNA melting profiles were analyzed by LightScanner instrument (Idaho Technologies). Melting curves were analyzed with the help of the LighScanner software and converted into appropriate segregation codes. For the gel separated markers, polymorphic marker alleles were recorded considering presence and absence.

The band and HRM records were compiled according to the genotype codes of population type CP described in the Joinmap^®^ 4 manual [50]. A consensus map was constructed with Kosambi’s mapping function following Joinmap^®^ 4 manual [50].

A comparative COSII map between the integrated potato genetic map, the potato physical map and the tomato genetic map was made as described in the legend to Figure 1. The figure was prepared using the genoPlotR library [51] for the statistical software R [52].

### Phylogenetic analysis

We ran a BLASTn against the DM genes and coding sequences provided by PGSC and the tomato genomic and coding sequences using our marker DNA sequences as queries. The marker sequences and the corresponding gene or coding sequences were aligned as DNA or translated amino acid sequences depending on whether the marker sequence obtained was covering intron or exon regions of the genes analyzed. The alignments were made using ClustalW and Neighbor Joining (NJ) trees were constructed using the Poisson correction method for amino acid sequences and the Maximum Composite Likelihood method for DNA sequences. Evolutionary analyses were conducted in MEGA5 [53].

### Ontology term annotation analysis

In the initial phase of the project the list of ontology terms associated with the 2868 COSII markers was manually reviewed and filtered for genes with gene ontology annotations that may have a role in traits of interest like stress tolerance and late blight resistance. For the final analysis, other criteria included single-copy status, and mapped in DM/DI//DI. This final list of 273 markers (further referred to as COSII-DM list) was subjected to the ‘Singular Enrichment Analysis’ tool as available on the AgriGO web-site [54]. The method tests if particular terms are over-represented or different in the set of interest against a reference list. We tested if the COSII-DM list was different from the original COSII list and versus the Arabidopsis gene model (TAIR9) as available on the AgriGO web-site. The focus of interest for the term analysis was on GO terms within the ‘biological process’ category.

## Competing interests

The authors declare that they have no competing interests.

## Authors’ contributions

HLK: Conducted *in silico* mapping and phylogenic analysis, led writing of paper. KC: Compiled sequence data and conducted phylogenetic analysis for earlier versions of the manuscript. LP: Selected markers on QTL intervals, conducted genetic mapping in potato and amplification of COS for all species, compiled sequence data. FR: Selected markers for analysis, coordinated generation of sequences from amplification products, helped write paper. RS: Supported selection of COS and analysed ontology. LM: Obtained funding, helped write paper, submitted sequences. DS: Obtained funding, helped write paper. MB: Obtained funding, helped write paper. All authors read and approved the final manuscript.

## Supplementary Material

Additional file 1: Table S1 Summary of all COS markers utilized in this study showing the marker locations in the DM genome, in the consensus potato genetic maps, and in TomEXPEN genetic map. The markers that were selected based on co-localization with the QTL for late blight resistance, maturity, ascorbic acid synthesis and carotenoid synthesis are shown in their corresponding columns, together with the literature reference. Also the PCR primer sequences for each marker are shown. Click here for file

Additional file 2: Table S3 Genetic linkage maps of the populations BCT, PCC1 and PD. For BCT all 12 linkage groups are shown, while for the other populations only the linkage groups where COS markers were mapped are shown. The denotation on the top of each linkage group indicates the name of the population. The markers are on the left of the groups and the cumulative distance in cM on the right. Click here for file

Additional file 3: Table S2 Single copy COS markers and the corresponding DM gene hits with putative functions and co-localization with the QTL for late blight resistance, maturity, ascorbic acid synthesis and carotenoid synthesis. Click here for file

Additional file 4: Figure S1 Graph of GO terms in the biological process category that are significantly enriched between the COSII-DM list and the TAIR9 gene model list. The Singular Enrichment Analysis (SEA) tool on the AgriGO website creates colors for nine significance levels. White corresponds to no difference (lowest level); yellow to the first level, light orange to the second level. The graph highlights that overall terms are slightly differently enriched and group into two broad categories: a) cellular metabolic processes and b) response to stimulus. Click here for file
