# Supplementary figures and images for: Linking the potato genome to the conserved ortholog set (COS) markers
Source: BMC Genet. 2013 Jun 8;14:51. doi: 10.1186/1471-2156-14-51 (PMC3691714; doi:10.1186/1471-2156-14-51)

## Slide 1
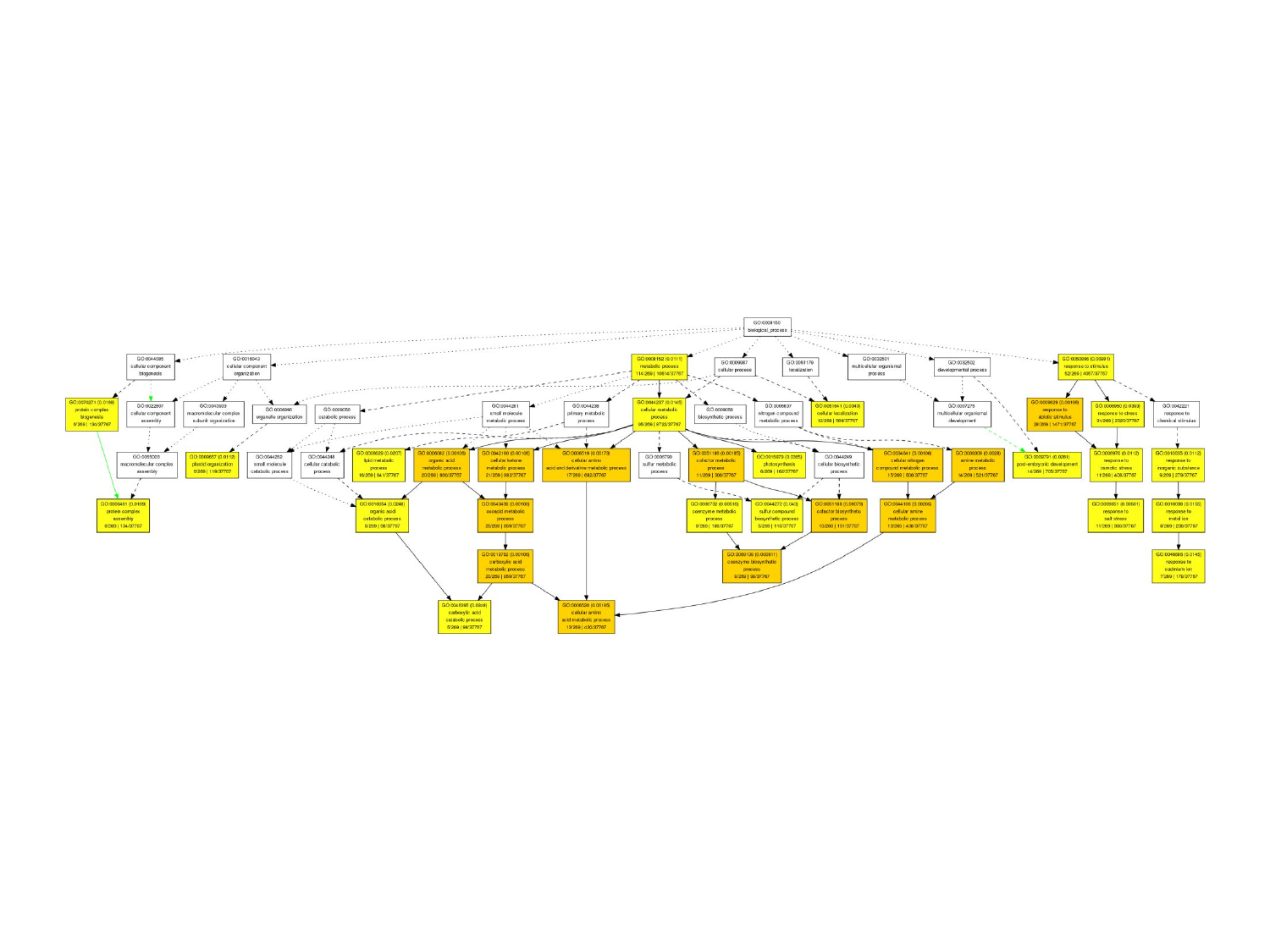

Supplement: Additional file 4: Figure S1 — Graph of GO terms in the biological process category that are significantly enriched between the COSII-DM list and the TAIR9 gene model list. The Singular Enrichment Analysis (SEA) tool on the AgriGO website creates colors for nine significance levels. White corresponds to no difference (lowest level); yellow to the first level, light orange to the second level. The graph highlights that overall terms are slightly differently enriched and group into two broad categories: a) cellular metabolic processes and b) response to stimulus. [file 1471-2156-14-51-S4.pptx]
